# Spatio-Temporal Distribution Characteristics and Trajectory Similarity Analysis of Tuberculosis in Beijing, China

**DOI:** 10.3390/ijerph13030291

**Published:** 2016-03-07

**Authors:** Lan Li, Yuliang Xi, Fu Ren

**Affiliations:** 1 School of Resource and Environmental Sciences, Wuhan University, 129 Luoyu Rd., Wuhan 430079, China; lilaninblue@whu.edu.cn (L.L.); yuliangwhu@163.com (Y.X.); 2 Key Laboratory of GIS, Ministry of Education, Wuhan University, 129 Luoyu Rd., Wuhan 430079, China; 3 Key Laboratory of Digital Mapping and Land information Application Engineering, National Administration of Surveying, Mapping and Geoinformation, Wuhan University, 129 Luoyu Rd., Wuhan 430079, China; 4 Collaborative Innovation Center of Geospatial Technology, Wuhan University, 129 Luoyu Rd., Wuhan 430079, China

**Keywords:** tuberculosis (TB), spatial autocorrelation, scan statistics, trajectory similarity, Beijing

## Abstract

Tuberculosis (TB) is an infectious disease with one of the highest reported incidences in China. The detection of the spatio-temporal distribution characteristics of TB is indicative of its prevention and control conditions. Trajectory similarity analysis detects variations and loopholes in prevention and provides urban public health officials and related decision makers more information for the allocation of public health resources and the formulation of prioritized health-related policies. This study analysed the spatio-temporal distribution characteristics of TB from 2009 to 2014 by utilizing spatial statistics, spatial autocorrelation analysis, and space-time scan statistics. Spatial statistics measured the TB incidence rate (TB patients per 100,000 residents) at the district level to determine its spatio-temporal distribution and to identify characteristics of change. Spatial autocorrelation analysis was used to detect global and local spatial autocorrelations across the study area. Purely spatial, purely temporal and space-time scan statistics were used to identify purely spatial, purely temporal and spatio-temporal clusters of TB at the district level. The other objective of this study was to compare the trajectory similarities between the incidence rates of TB and new smear-positive (NSP) TB patients in the resident population (NSPRP)/new smear-positive TB patients in the TB patient population (NSPTBP)/retreated smear-positive (RSP) TB patients in the resident population (RSPRP)/retreated smear-positive TB patients in the TB patient population (RSPTBP) to detect variations and loopholes in TB prevention and control among the districts in Beijing. The incidence rates in Beijing exhibited a gradual decrease from 2009 to 2014. Although global spatial autocorrelation was not detected overall across all of the districts of Beijing, individual districts did show evidence of local spatial autocorrelation: Chaoyang and Daxing were Low-Low districts over the six-year period. The purely spatial scan statistics analysis showed significant spatial clusters of high and low incidence rates; the purely temporal scan statistics showed the temporal cluster with a three-year period from 2009 to 2011 characterized by a high incidence rate; and the space-time scan statistics analysis showed significant spatio-temporal clusters. The distribution of the mean centres (MCs) showed that the general distributions of the NSPRP MCs and NSPTBP MCs were to the east of the incidence rate MCs. Conversely, the general distributions of the RSPRP MCs and the RSPTBP MCs were to the south of the incidence rate MCs. Based on the combined analysis of MC distribution characteristics and trajectory similarities, the NSP trajectory was most similar to the incidence rate trajectory. Thus, more attention should be focused on the discovery of NSP patients in the western part of Beijing, whereas the northern part of Beijing needs intensive treatment for RSP patients.

## 1. Introduction

Tuberculosis (TB) is a chronic infectious disease caused by Mycobacterium tuberculosis [1]. TB is primarily transmitted through the respiratory tract, and TB patients serve as the main source of infection. TB is also an important public health issue in China [2]; the fifth national sample survey of epidemic diseases conducted in 2010 showed that reported TB patients always formed the front rank of reported cases of national Class A and B epidemic diseases [3]. Considerable pressure has built for the prevention and control of TB, especially in cities because of rapid urbanization and economic transition [4].

Most epidemic data have spatial attributes, which has aroused the interest of researchers with both medical and geographic backgrounds. With the development of Geographic Information System (GIS) technology and its application in epidemiology, a new branch of epidemiology called spatial epidemiology has formed. Spatial epidemiology uses spatial information to extend the analysis of epidemic diseases [5]. With the aid of spatial statistics and mapping visualization, spatial epidemiology attempts to describe and analyse the spatial distribution of human diseases, health conditions and latent factors [6,7,8,9,10,11]. Spatial epidemiology also explores the spatial distribution model to predict the spatio-temporal trends of disease and the correlation between a disease and its latent factors [12,13,14]. All of this information can be used to monitor and prevent disease, process outbreaks and allocate medical resources in an evidence-based manner [15].

Disease mapping can present distributions intuitively. This method was first used in the investigation of a cholera outbreak caused by street water pump pollution in London in 1854. However, it explores disease only qualitatively. Quantitative methods, such as spatial autocorrelation, can objectively describe the distribution characteristics of disease, and scan statistics can describe the clustering characteristics in terms of both space and time [16,17,18]. Analysing the clustering characteristics of a disease can help detect hot spots and high-risk groups in space and time, which in turn helps decision makers formulate specific prevention and treatment policies [19].

Trajectory data contain the sequence of location and time information for moving objects. Trajectory similarity analysis has been used in fields such as transport logistics, human behaviour, and marketing management [20]. From the perspective of spatial epidemiology, the changing time sequence of spatial statistics, such as the mean centre, nearest-neighbour distance and spatial correlation coefficient, can be regarded as trajectory data [21,22]. New smear-positive (NSP) TB indicates new disease occurrence, whereas retreated smear-positive (RSP) TB is more resistant to drugs. Because both types of TB are infectious, it is necessary to analyse the distribution difference between the overall incidence rates of TB and the incidence rates for these two categories [23]. The analyses can also reflect variations in the control effect among districts and loopholes in prevention strategies. The current study applied the Euclidean distance algorithm to measure the similarities between trajectories over the full time interval. However, because we were interested in the occurrence of disease close to the current year, weights were assigned to different times to modify the algorithm.

The present study aimed to clarify the spatial and temporal distribution characteristics of TB at the district level in Beijing, China from 2009 to 2014. We utilized spatial statistics, spatial autocorrelation and scan statistics analyses to describe the distribution characteristics and clustering characteristics of TB. We evaluated the trajectory similarities between TB and NSP TB/RSP TB over a five-year period with the intent of detecting the differential distribution of these three categories and the similarity variations over time. The study investigated (1) the spatial and temporal trends in TB from 2009 to 2014; (2) the global spatial autocorrelation of overall TB across the districts of Beijing and the local spatial autocorrelation of TB in individual districts of Beijing; (3) the purely spatial, purely temporal and spatio-temporal clusters and variations of TB; and (4) trends in the mean centres of TB, NSP TB and RSP TB and the trajectory similarities of TB and NSP TB/RSP TB over a five-year period.

## 2. Materials and Methods

### 2.1. Study Area

**Figure 1:**
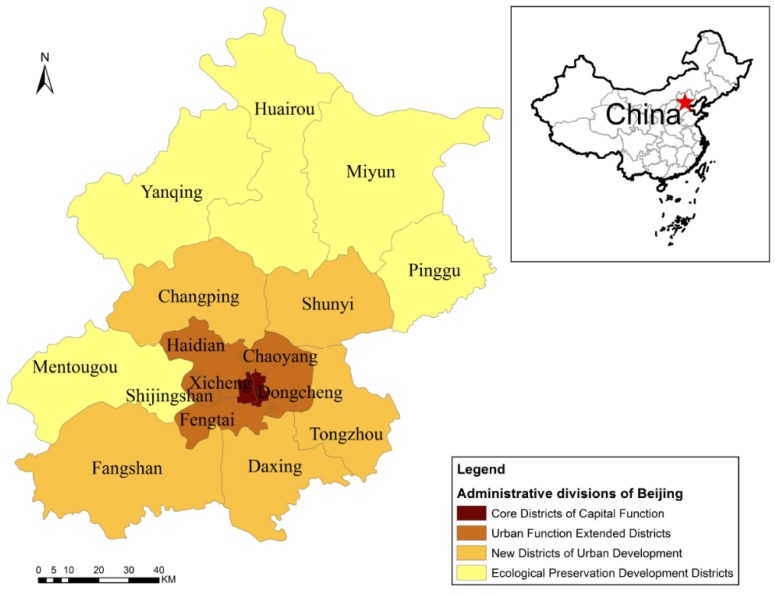
Map of the administrative districts of Beijing according to their different urban functions and their locations in China.

Beijing (the capital of China) is the nation’s political, cultural centre and economic centre. Located in the northwestern portion of the North China Plain, Beijing is adjacent to Tianjin on the southeast and Hebei province in the other directions. Beijing was divided into 16 administrative districts in July 2010, and these districts are still in place today. According to their different urban functions, these districts can be classified into four regions: the Core Districts of Capital Function (Dongcheng and Xicheng), the Urban Function Extended Districts (Chaoyang, Fengtai, Shijingshan, and Haidian), the New Districts of Urban Development (Fangshan, Tongzhou, Shunyi, Changping, and Daxing), and the Ecological Preservation Development Districts (Mentougou, Huairou, Pinggu, Miyun, and Yanqing) (Figure 1). The study area included the 16 districts under the administration of Beijing according to the latest administrative divisions.

### 2.2. Data Description

#### 2.2.1. TB Data

The TB data for each administration district from 2009 to 2014 were obtained from the Beijing Health and Population Health Status Reports [24,25,26,27,28]. These data were published by the Beijing Centre for Disease Prevention and Control to provide open medical and health information. These data include the number of NSP TB patients, the number of RSP TB patients, the number of smear-negative TB patients, the number of TB patients who did not receive a sputum smear examination, the number of TB pleurisy patients and the total number of TB patients for each administrative district. Because China has only recently begun to share medical data with the public, we only had access to six years of data. However, the data from 2009 only provide the total number of TB patients and does not include detailed information about the number of NSP, RSP and other categories of TB patients. Therefore, except in the trajectory similarity analysis, we used the data from 2009 to 2014. In the trajectory similarity analysis, we used the data from 2010 to 2014 because the 2009 data are not detailed enough to be suitable for this analysis. The TB data were available at the temporal resolution of year and at the spatial resolution of administrative district.

#### 2.2.2. Population Data

The annual population data for each administrative district from 2009 to 2014 were obtained from the Beijing Statistical Information Net [29]. We used the resident population that had lived in Beijing for more than six months of the last year as the population for this study.

#### 2.2.3. Spatial District Data

Fundamental geographic data with a scale of 1:4,000,000 were obtained from the National Geomatics Centre of China [30]. To display the spatial distribution of TB and to perform the spatial analysis, the TB data and population data of each administrative district were imported into the attribute table of spatial district data. The data were double-checked to prevent errors. We used the administrative centre of each district to represent each district in the scan statistics analysis, and the longitude and latitude of the centres were used in trajectory similarity analysis. Because we assumed that more people lived and worked near the city than in the suburbs, especially in a cosmopolitan city such as Beijing, we used the administrative centres to better reflect the centre of human activities for each district. The longitude and latitude of the centres are available from the network [31].

### 2.3. Methodology

#### 2.3.1. Registration Rate and Incidence Rate Calculations

The registration rate (*rr*) is expressed as the observed and registered number of TB patients per 100,000 residents using the total population of the corresponding district as the standard. This rate can be described as follows:

(1) $$rr_{i}=\frac{O_{i}}{N_{i}}\times 100，000$$

where $O_{i}$ and $N_{i}$ are the total number of TB patients (including the number of NSP TB patients, the number of RSP TB patients, the number of smear-negative TB patients, the number of TB patients who did not undergo a sputum smear examination, and the number of TB pleurisy patients) and the total population in the *i*th district per year, respectively. The incidence rate (IR) is used to represent the disease risk across Beijing in this study, to identify districts with higher or lower disease risks and to capture the temporal and spatial clusters. In 2010, Beijing provided TB patients with free examinations for the first time and fully covered directly observed treatment + short-course chemotherapy (DOTS) [32]. Therefore, in this study, we postulated that the IR approximately equalled the *rr*.

#### 2.3.2. Spatial Autocorrelation Analysis

All attribute values on a geographic surface are related to one another, but closer values are more strongly related than more distant values [33]. The spatial autocorrelation analysis by GeoDa (Arizona State University, Phoenix, AZ, USA) is used to test whether there is interdependence and to determine the level of interdependence between the same attribute values of one spatial unit and its neighbouring units [34]. Global spatial autocorrelation analysis reflects the autocorrelation across the whole area but cannot reflect the local distribution characteristics of the attribute value and its contribution to the global autocorrelation. Therefore, local spatial autocorrelation analysis is used to detect the autocorrelation of each spatial district and its variation across the area.

Constructing a spatial weight matrix that reflects the spatial adjacent correlation among spatial districts is the first step in the spatial autocorrelation analysis [35]. In this study, we used the binary spatial weight matrix, which is the most commonly used matrix in practice. It can be described as follows:

(2) $$W_{ij}=\{\begin{array}{cc} 1 & A_{i}\;is\;adjacent\;to\;A_{j}\\ 0 & others \end{array}$$

where $W_{ij}$ is the element of the spatial weight matrix reflecting the spatial adjacent correlation between different spatial districts $A_{i}$ and $A_{j}$. When $A_{i}$ shares common boundaries with $A_{j}$, $W_{ij}$ equals 1. Otherwise, $W_{ij}$ equals 0, including the condition in which $A_{i}$ shares no common boundaries with $A_{j}$ and when *i* equals *j*. 

The most commonly used statistic to measure autocorrelation is Moran’s *I.* The global Moran’s *I* statistic ranges from −1 to 1. I>0 indicates positive autocorrelation. I<0 indicates negative autocorrelation. I=0 indicates no autocorrelation. When |I| is larger, the autocorrelation is higher. The global Moran’s *I* can be calculated as follows [5]:

(3) $$I=\frac{n\cdot \sum_{i=1}^{n}\sum_{j=1}^{n}W_{ij}\left(y_{i}-\bar{y}\right)\left(y_{j}-\bar{y}\right)}{\sum_{i=1}^{n}{\left(y_{i}-\bar{y}\right)}^{2}\cdot \sum_{i=1}^{n}\sum_{j=1}^{n}W_{ij}}$$

where n is the number of districts, $y_{i}$ and $y_{j}$ are the IR values of spatial districts *i* and *j*, $\bar{y}$ is the average IR of all districts, and $W_{ij}$ is the element of the spatial weight matrix corresponding to the district pair *i* and *j*.

The local Moran’s *I* satisfies two conditions: the local indication of spatial autocorrelation (LISA) reflects the clustering level between one spatial district and its neighbouring districts, and the sum of all LISA values is proportional to the global Moran’s *I*. There are four spatial correlation modes: the spatial district with a high IR surrounded by districts with high IR values (High-High); the spatial district with a low IR surrounded by districts with high IR values (Low-High); the spatial district with a low IR surrounded by districts with low IR values (Low-Low); and the spatial district with a high IR surrounded by districts with low IR values (High-Low). For spatial district *i*, LISA can be calculated as follows:

(4) $$I_{i}=\frac{\left(y_{i}-\bar{y}\right)}{\frac{1}{n}\sum_{i=1}^{n}{\left(y_{i}-\bar{y}\right)}^{2}}\times \sum_{j=1}^{n}W_{ij}\left(y_{i}-\bar{y}\right)$$

A larger $\left|I_{i}\right|$ indicates higher clustering level in the *i*th district. If $I_{i}$ is positive, the *i*th district is the area where the lever of incidence is similar to the surrounding areas (High-High or Low-Low). In contrast, if $I_{i}$ is negative, the *i*th district is the area that is dissimilar to the surrounding areas (High-Low or Low-High). If $\left|I_{i}\right|$ is close to 0, the occurrence of TB is randomly distributed, and there is no clustering phenomenon.

#### 2.3.3. Scan Statistics Analysis

Scan statistics analysis, a retrospective statistical test based on a discrete Poisson model performed by SaTScan (Martin Kulldorff, Boston, MA, USA), is used to detect whether the IR of TB shows clustering characteristics and to determine the location and relative risk (RR) of the clusters. A pure spatial scan statistics analysis is defined by a circular window with a radius that varies continuously according to the population range of the area. The radius moves throughout the study area to detect several cluster centroids from zero to the maximum cluster size of the total population that might be at risk. Purely temporal scan statistics analysis is similar to the purely spatial scan statistics analysis; however, the scan range is a time period. Space-time scan statistics analysis incorporates the time dimension and is defined by a cylindrical window with a geographic base and a height corresponding to time [36]. In this study, the default maximum spatial cluster size of 50% was selected for the cluster analysis. Furthermore, the log likelihood ratio (LLR) was used to calculate the difference in the incidence inside and outside the windows [37]:

(5) $$LLR=\text{log}{\left(\frac{O_{in}}{E_{in}}\right)}^{O_{in}}{\left(\frac{O-O_{in}}{O-E_{in}}\right)}^{\left(O-O_{in}\right)}$$

where $O_{in}$ and $E_{in}$ denote the numbers of actual and expected cases in the window, respectively. $E_{in}$ is calculated by multiplying the general IR of Beijing by the population of the *i*th district and can be expressed as follows:

(6) $$E_{i}=G_{IR}\times N_{i}$$

where $N_{i}$ is the total population of the *i*th district. $G_{IR}$ is the general IR of Beijing, which can be described as follows:

(7) $$G_{IR}=\frac{\sum_{j=1}^{n}O_{j}}{\sum_{j=1}^{n}N_{j}}$$

where *n* is the number of districts administered by Beijing, and $O_{j}$ and $N_{j}$ are the number of observed cases and the population in the *j*th (j=1,2,…,n) district, respectively. The most likely cluster is the scan window with the largest LLR value, and the secondary clusters are the other scan windows with significant LLR values. The TB patients and populations of each district in each year and the coordinates of each district were included to obtain the most likely cluster in which the districts and time frame had the largest LLR and the maximum RR.

#### 2.3.4. Trajectory Similarity Analysis

The mean centre (MC) tool in ArcGIS (Environmental Systems Research Institute, Redlands, CA, USA) was used to identify the spatio-temporal change in TB in Beijing from 2009 to 2014. The MC identifies the geographic centre of a set of points to measure the central tendency, which is calculated as follows:

(8) $$MC_{t}=\left(X_{t},Y_{t}\right)$$

(9) $$X_{t}=\frac{\sum_{j=1}^{n}IR_{j}\cdot x_{j}}{\sum_{j=1}^{n}IR_{j}}$$

(10) $$Y_{t}=\frac{\sum_{j=1}^{n}IR_{j}\cdot y_{j}}{\sum_{j=1}^{n}IR_{j}}$$

where ${MC}_{t}$ denotes the coordinates of the MC in the *t*th (t=1,2,…,m) year, *n* is the number of points over the study area in the *t*th year, and $x_{j}$ and $y_{j}$ are the coordinates of the *j*th (j=1,2,…,n) point in the *t*th year. The IR MCs of the same geographic area in a time series could reveal the movement of the IR central tendency. The MCs of NSP TB patients in the resident population (NSPRP), NSP TB patients in the TB patient population (NSPTBP), RSP TB patients in the resident population (RSPRP) and RSP TB patients in the TB patient population (RSPTBP) from 2010 to 2014 were calculated to identify and compare the yearly movement of the central tendency.

A trajectory is a serial record of spatial locations of moving objects with time attributes. The central tendencies of IR, NSPRP, NSPTBP, RSPRP and RSPTBP over time can be regarded as a type of trajectory. The Euclidean distance between different tracks can be used to measure the trajectory similarities of IR and the other four categories’ central tendencies. The Euclidean distance between tracks is based on the Euclidean distance between points. First, the distance between points is calculated using the same time, and the sum of these distances is then calculated. The Euclidean distance of two tracks can be calculated as follows:

(11) $$dist(MC1,MC2)=\sum_{t=1}^{m}p_{t}\cdot dist(MC1_{t},MC2_{t})$$

where dist(MC1,MC2) is the Euclidean distance of two different tracks MC1 and MC2. When the distance is smaller, the trajectory similarity between them is higher. The total points of each track are the same and equal m. $p_{t}$ is the weight for different point pairs according to certain rules. Because the situation is very similar for adjacent years, we assume that the IR of current year is more strongly correlated with the IR of the previous year than with that of the year before last, three years ago and so on. Therefore, because we focused on the IR close to the current year, we assigned a greater weight to the most recent year. For exponential function, a should meet the condition a>0 and a≠1. When 0<a<1, the curve of trend component is a monotonically decreasing function; when a>1, the curve of trend component is a monotonically increasing function. Therefore, we used the exponential function model (0<a<1) to give the point pairs different weights and unitized the results, which can be expressed as follows:

(12) $$p_{t}=\frac{{\left(a\right)}^{k}}{\sum_{k=0}^{m-1}{\left(a\right)}^{k}}\;\;(0<a<1)$$

where k=0,1,2⋅⋅⋅m-1 starting from the current year. Because the curve is characterized by a sharper slope when a is close to 0. If a were small, the weights for years far from current year would be too small. Therefore, we assigned 0.5≤a<1. We generated 1000 random numbers between 0.5 and 1, than performed point estimation in the sample, and the result showed a=0.75. Therefore, we established five gradient values for a (a=0.55, a=0.65, a=0.75, a=0.85 and a=0.95) which were symmetrical by 0.75. However, the number of gradient values is not fixed as long as the selected numbers are symmetrical by 0.75. $MC1_{t}$ and $MC2_{t}$ are the point pairs of the track MC1 and the track MC2 in the *t*th year, respectively. $dist(MC1_{t},MC2_{t})$ is the Euclidean distance between the point pairs $MC1_{t}$ and $MC2_{t}$, which can be calculated as follows:

(13) $$dist(MC1_{t},MC2_{t})=\sqrt{{\left(X1_{t}-X2_{t}\right)}^{2}+{\left(Y1_{t}-Y2_{t}\right)}^{2}}$$

where $X1_{t}$ and $X2_{t}$ can be calculated using Equation (9) and $Y1_{t}$ and $Y2_{t}$ can be calculated using Equation (10). The unit of distance is the kilometre (km).

## 3. Results and Discussion

### 3.1. Spatio-Temporal Distribution

**Figure 2:**
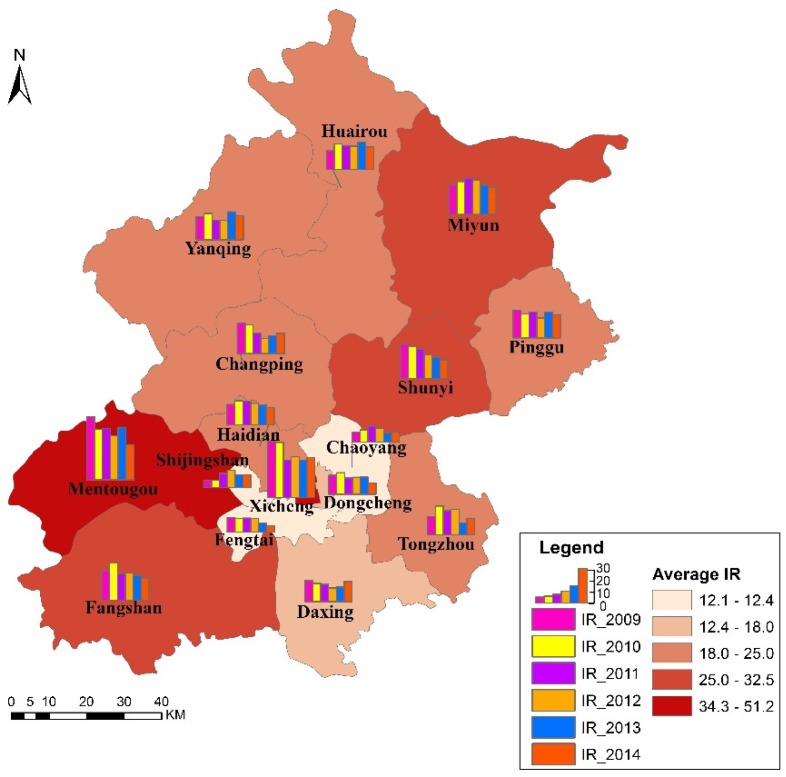
Map of the IRs and average IRs of TB from 2009 to 2014 at the district level in Beijing.

**Figure 3:**
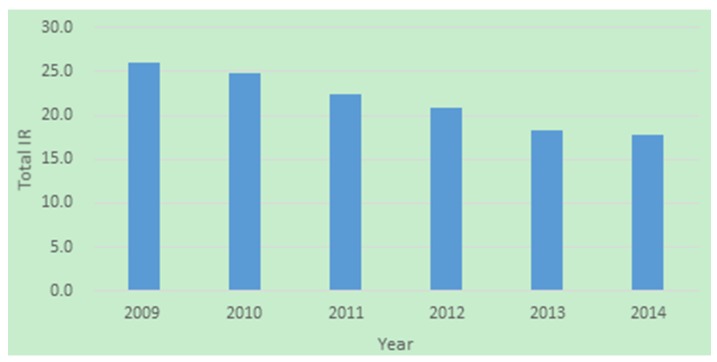
The average IRs of TB overall across the districts of Beijing from 2009 to 2014.

The temporal distribution of IRs for each district in Beijing from 2009 to 2014 is shown in Figure 2. The colour of each district represents the average IR related to TB from 2009 to 2014, and the bar charts illustrate the annual IR from 2009 to 2014 (*i.e.*, IR_2009 to IR_2014) for each district. We used the natural break method to appropriately cluster similar values from a set of data and to maximize the gap between groups to classify the IRs into five groups. The largest average IRs of TB were in Mentougou (51.0/100,000) and Xicheng (45.5/100,000); the next largest average IRs of TB were in Miyun (32.5/100,000), Fangshan (28.9/100,000) and Shunyi (27.7/100,000). The mid-range average IRs of TB were in Pinggu (25.0/100,000), Huairou (24.4/100,000), Yanqing (24.1/100,000), Changping (22.8/100,000), Haidian (21.9/100,000) and Tongzhou (21.4/100,000). The second smallest average IRs of TB were in Daxing (18.0/100,000) and Dongcheng (17.6/100,000), and the smallest average IRs of TB were in Shijingshan (12.4/100,000), Fengtai (12.3/100,000) and Chaoyang (12.1/100,000). The average IRs of TB overall across the districts of Beijing from 2009 to 2014 (Figure 3) showed a downward trend that was consistent with the IRs of TB’s overall downward trend in China since 2008. The total average IR of TB overall across the districts of Beijing over six years was 21.7/100,000, which was lower than the average IR of TB in China as a whole. The first time that the average IR of TB overall across the districts of Beijing was smaller than 20/100,000 was in 2013. We performed two GLM analyses on the six-year data using SPSS (IBM, Armonk, NY, USA). One compared if TB IRs have difference in different years (looking for temporal trend), and the other compared if TB IRs have difference if different districts (looking for spatial trend). In the first analysis, we considered year (six years from 2009 to 2014) as the fix factor. The results showed that IRs for different years have no difference (*p* = 0.528). In the latter analysis, we considered district (16 districts in Beijing totally) as the fix factor. The results showed that IRs for different districts have difference (*p* = 0.000). Sixteen districts in Beijing were divided into five subsets: (1) Chaoyang, Fengtai, Shijingshan, Dongcheng and Daxing; (2) Dongcheng, Daxing, Tongzhou, Haidian, Changping, Yanqing, Huairou and Pinggu; (3) Tongzhou, Haidian, Changping, Yanqing, Huairou, Pinggu, Shunyi and Fangshan; (4) Yanqing, Huairou, Pinggu, Shunyi, Fangshan and Miyun; (5) Xicheng and Mentougou. IRs in the same subset have no statistical significance and IRs in different subsets have statistical significance. All analyses were with the significance level α<0.05.

The cause of the decline in the IRs from 2009 to 2014 may be that the Chinese government has made great efforts to prevent and control TB [38]. The State Council issued documents concerning the National Tuberculosis Control Program (2001–2010) and the National Tuberculosis Control Program (2011–2015) [39], and implemented the DOTS policy at the county level. These policies can detect the source of infection abundantly and directly, can often cure newly discovered patients without the need for hospitalization, can alleviate the financial burden on patients because it costs much less, and can decrease the occurrence of drug-resistant TB [40]. DOTS has made remarkable achievements and has effectively suppressed the increasing tendency of TB. The Chinese government has delivered many types of education and awareness activities (especially on World Tuberculosis Day every year) to improve public awareness of TB prevention. Beijing, which is the nation’s political, cultural and economic centre, has good economic and medical conditions. Since 1979, Beijing has provided free treatment to a portion of TB patients every year. In recent years, the Beijing government expanded the range of free treatments to include TB patients who were non-permanent residents for the first time and alleviated the inspection charge to a certain extent. Moreover, in addition to the Beijing Research Institute of Tuberculosis Control, there are TB prevention and control institutes in each district that provide TB patients with convenient treatment. By expanding its prevention and control network, Beijing deepened the prevention and control responsibilities of the community service centres to strengthen infection control efforts.

### 3.2. Spatial Autocorrelation Analysis

The result shows that the global Moran’s *I* (−0.1198) was negative and failed to pass the significance level test (*p* = 0.44), indicating that there was no global spatial autocorrelation and that the occurrence of TB was distributed randomly from 2009 to 2014.

**Figure 4:**
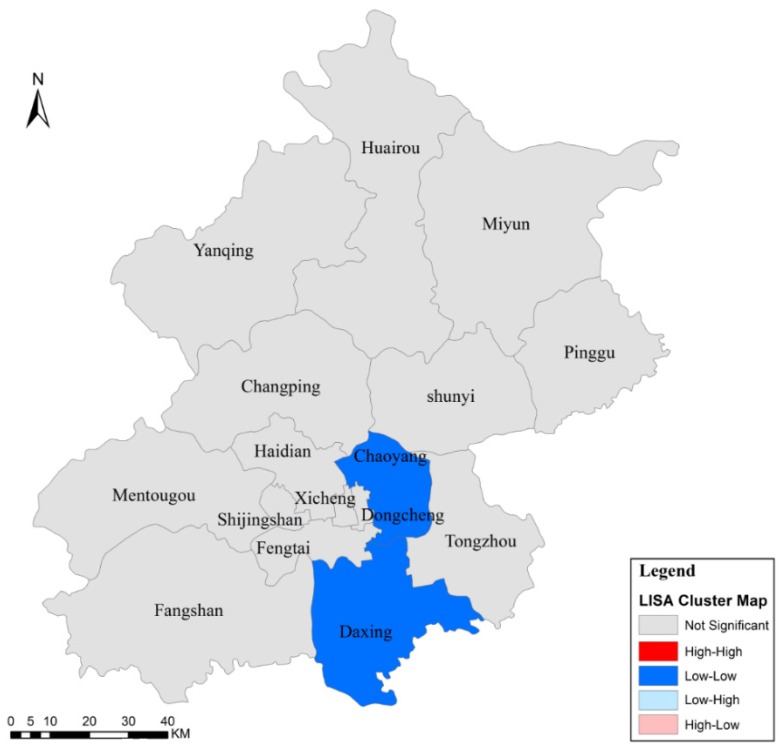
LISA cluster map of Beijing from 2009 to 2014.

The results of the local autocorrelation analysis are mapped in Figure 4. The LISA cluster map shows that the Chaoyang and Daxing districts were Low-Low districts over the six-year study period, indicating that the IRs of Chaoyang and Daxing were low and the IRs of their neighbouring districts were also low. Other districts (in grey) did not show any local spatial autocorrelation characteristics. The results all passed significance level testing (*p* < 0.05).

From these results, we might surmise that the disease was not spreading in Chaoyang and Daxing because there was no influx of infection from their neighbouring districts. The overall occurrence of TB across the districts of Beijing was relatively low and steady over the six-year period, and there were no outbreaks, indicating that the efforts of the Beijing government were successful.

### 3.3. Scan Statistics Analysis

The investigation of the purely spatial scan statistics analysis of high and low IR at the district level (Table 1) revealed that the first rank cluster contained only the Xicheng district, which had a high IR. The second rank cluster included one low IR district: Chaoyang. The third rank cluster contained two low IR districts: Shijingshan and Fengtai. The fourth and fifth rank clusters had high IRs: the fourth contained Mentougou, and the fifth contained Miyun, Huairou, Shunyi and Pinggu. All of the results passed significance level testing (*p* = 0.001).

**Figure 5:**
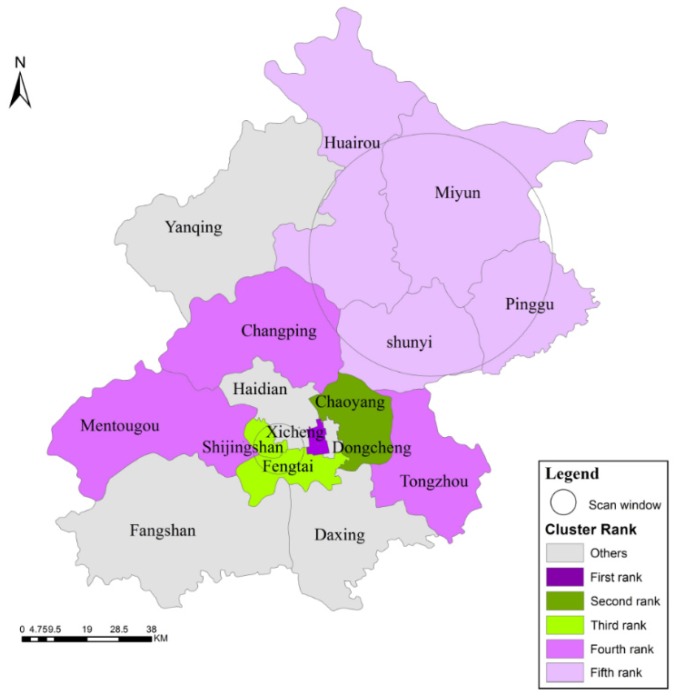
High and low IR spatial clusters in Beijing from 2009 to 2014.

The distribution of high and low IR spatial clusters in Beijing from 2009 to 2014 is showed in Figure 5. The Xicheng district ranked first, with a high IR over the six-year period. One possible explanation is that Xicheng had a large population and a high population density, resulting in crowded living conditions, bad air ventilation and poor sanitary conditions, which all contribute to TB transmission. The growth of floating populations in Xicheng was not significant, but it was the district with the densest floating population. Moreover, the majority of the floating population in this district engaged in the commercial service industry, which was liquid and offered a greater chance for contact with people. Once TB gained a foothold, the disease could be transmitted to the exposed population easily, leading to extensive transmission.

The second and third rank clusters were located in the Urban Function Extended Districts, including Chaoyang, Shijingshan and Fengtai. One possible explanation for this formation of distribution characteristics among the low-IR clusters is that although the Urban Function Extended Districts were the main clusters of the floating population (*i.e.*, the floating populations of Chaoyang, Haidian and Fengtai comprised more than 50% of the total floating population of Beijing, with a downward trend), the floating population of the New Districts of Urban Development showed apparent growth and trended toward movement from the Urban Function Extended Districts to the New Districts of Urban Development (*i.e.*, the floating populations of Daxing, Tongzhou, Changping and Shunyi comprised more than 20% of the total floating population of Beijing, with an upward trend). Furthermore, the floating population of the Urban Function Extended Districts primarily comprised fixed groups, such as students and construction workers, who live in certain areas with a limited scope of communication activities. This phenomenon decreases both the chances for individuals to contact others and the possibility of transmission.

The investigation of the purely temporal scan statistics analysis of high and low IR at the district level (Table 2) revealed that there was only one significant cluster. The first rank cluster showed that the IR was high over the three-year period from 2009 to 2011. The result was consistent with the downward trend in the IR over the six-year study period. The result passed significance level testing (*p* = 0.001).

**Figure 6:**
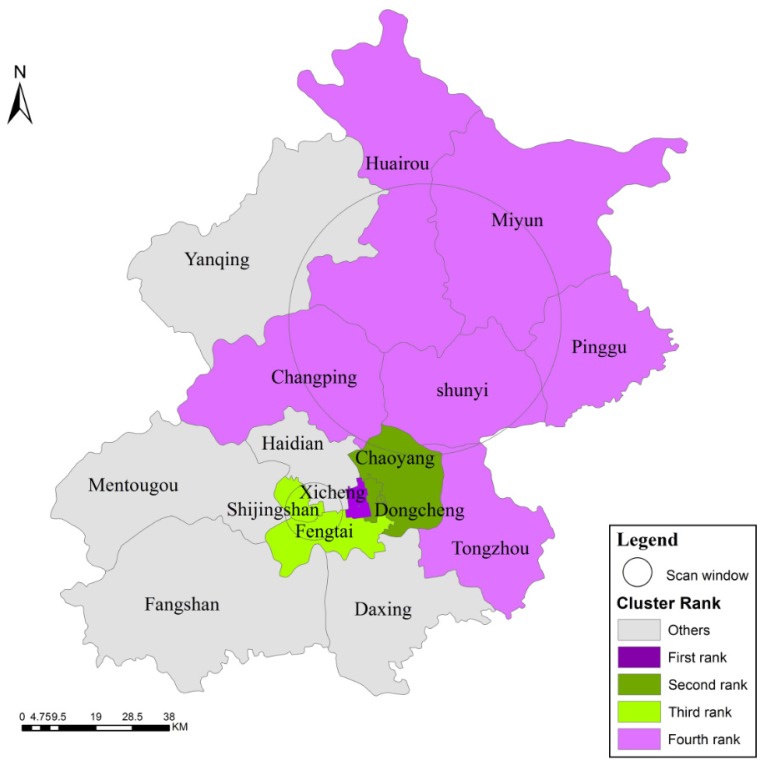
High and low IR spatio-temporal clusters in Beijing from 2009 to 2014.

The space-time scan statistics analysis of high and low IRs (Table 3) revealed four significant clusters. The first rank cluster contained only the Xicheng district, which had a high IR from 2009 to 2011, whereas the second rank cluster contained two districts with low IRs, Dongcheng and Chaoyang, from 2012 to 2014. The third rank cluster included Shijingshan and Fengtai, with low IRs from 2012 to 2014. The fourth rank cluster contained four high IR districts: Huairou, Miyun, Shunyi and Changping (from 2009 to 2010). The distribution characteristics of the spatial clusters (Figure 6) were consistent with the results of the purely spatial scan statistics analysis. The temporal cluster results were consistent with the downward trend in the IR over the six-year period. All of the results passed significance level testing (*p* = 0.001).

### 3.4. Trajectory Similarity Analysis

**Figure 7:**
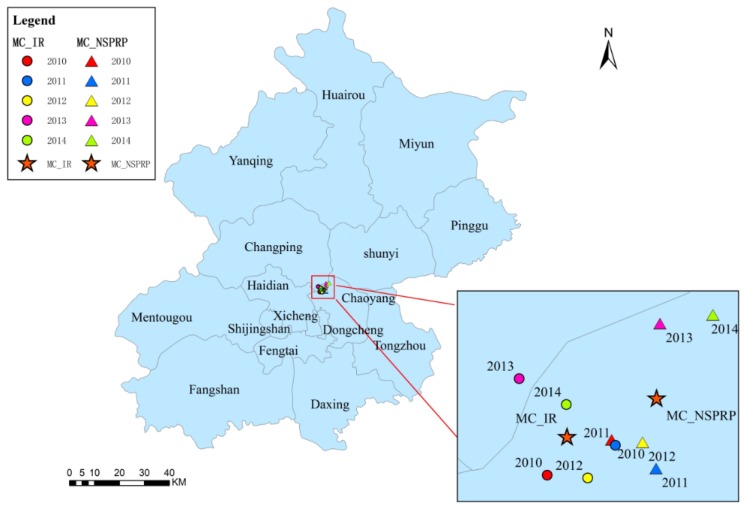
The distributions of IR and NSPRP MCs in Beijing from 2010 to 2014.

**Figure 8:**
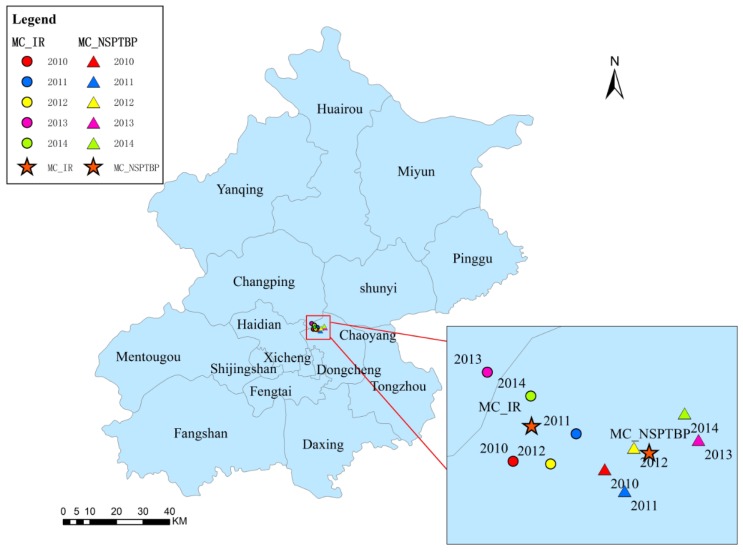
The distributions of IR and NSPTBP MC in Beijing from 2010 to 2014.

**Figure 9:**
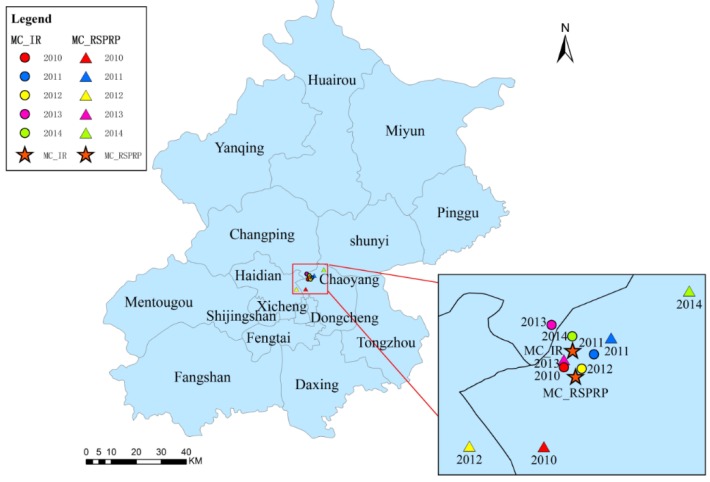
The distributions of IR and RSPRP MC in Beijing from 2010 to 2014.

**Figure 10:**
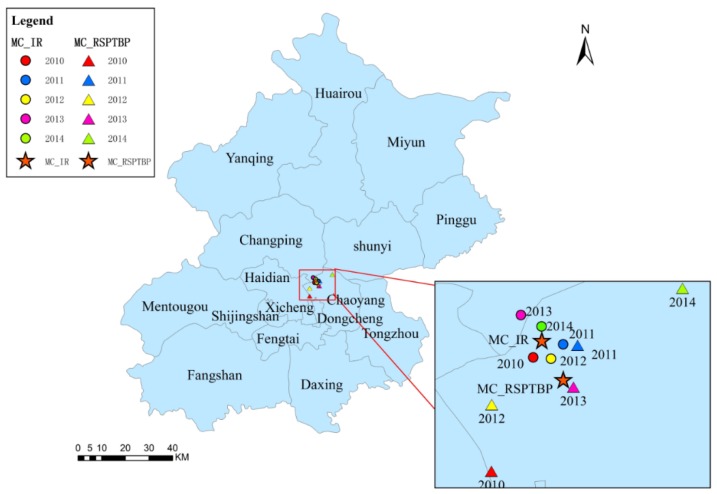
The distributions of IR and RSPTBP MC in Beijing from 2010 to 2014.

The MC analysis revealed that the majority of the MCs were in adjacent locations near the common boundaries of Chaoyang and Changping and Chaoyang and Haidian. The general distribution of the NSPRP MCs and NSPTBP MCs were to the east of the IR MCs (Figure 7 and Figure 8). The general distribution of the RSPRP MCs and RSPTBP MCs were to the south of the IR MCs (Figure 9 and Figure 10).

The results of the trajectory similarity analysis revealed that from 2010 to 2014, the similarity between the IR and NSPRP (2.70) was the greatest; the similarity between the IR and NSPTBP (3.41) followed next; the similarity between the IR and RSPRP (4.46) sorted third; the similarity between the IR and RSPTBP (5.06) sorted lowest (Table 4).

Because the NSP trajectory was the most similar to the IR trajectory and the NSP TB patients are newly discovered patients who could be latent and could transmit TB to others, more attention should be paid to the discovery of NSP TB patients in the western part of Beijing. Because RSP TB is still transmissible and has a greater possibility of becoming resistant to drugs, the southern part of Beijing may have a lower cure rate than the northern part; therefore, TB patients in the southern part require intensive treatment. Thus, health-related policies should be formulated to take measures to adjust these factors, and public health resources should be allocated more appropriately. By improving the identification of NSP TB patients and cure rate of RSP TB patients, the prevention of TB in Beijing would have better results.

## 4. Conclusions

This study investigated the distribution characteristics of spatio-temporal clusters of TB at the district level in Beijing from 2009 to 2014 using GeoDa and SaTScan software and ArcGIS tools; furthermore, it evaluated the trajectory similarities between IR and NSPRP/NSPTBP/RSPRP/RSPTBP over a five-year period to provide guidelines for the allocation of public health resources and to strengthen health-related policies. We found that the IRs of Beijing exhibited a gradual decrease from 2009 to 2014, possibly because of the Beijing government’s considerable efforts to prevent and control TB. Global spatial autocorrelation was not observed across all of the districts of Beijing, indicating that the occurrence of TB was randomly distributed. However, there was a local spatial autocorrelation at the district level, with Chaoyang and Daxing as the Low-Low districts over the six-year period. The scan statistics analysis showed spatial, temporal and spatio-temporal clusters of high and low IR. The distribution of MCs showed that the general distributions of NSPRP MCs and NSPTBP MCs were to the east of the IR MCs. Conversely, the general distribution of RSPRP MCs and RSPTBP MCs were to the south of the IR MCs. Based on the combined analysis of the MC distribution characteristics and trajectory similarities, the trajectory of NSP TB was most similar to the trajectory of IR. Thus, more attention should be focused on identifying NSP TB patients in the western part of Beijing, whereas the southern part of Beijing needs to offer intensive treatment for RSP TB patients. These results can be used by urban public health officials and related decision makers to allocate public health resources and to formulate prioritized health-related policies.

Because China has only recently begun to share medical data with the public, we only had access to six years of data. In addition, we only had the number of patients, but do not have access to more detailed information about patients such as their occupation, gender, age and so on. The TB data were available at the spatial resolution of administrative district. If we had the data at the spatial resolution of the subdistrict community, we could detect the distribution characteristics more specifically. This study only qualitatively analysed the causes of the TB distribution characteristics. Because of data limitations, it is hard to detect the latent factors leading to the distribution characteristics at this stage. Therefore, further studies are necessary to detect the risk factors. Furthermore, we used an exponential function to give the point pairs different weights. In future study, we would explore a more complicated and appropriate function to replace this basic function. These limitations will be investigated in the future.

## Figures and Tables

**Table 1:** Purely spatial scan statistics analysis of high and low IRs from 2009 to 2014.

Cluster Rank	IR	Number of Districts	Districts	Number of Cases	Expected Cases	*LLR*	*RR*	*p*
First	High	1	Xicheng	3458	1638.88	832.68	2.28	<0.001
Second	Low	1	Chaoyang	2685	4707.95	609.13	0.52	<0.001
Third	Low	2	Shijingshan, Fengtai	2078	3584.80	423.30	0.54	<0.001
Fourth	High	1	Mentougou	906	380.86	265.43	2.43	<0.001
Fifth	High	4	Miyun, Huairou, Shunyi, Pinggu	3640	2812.79	126.16	1.34	<0.001

**Table 2:** Purely temporal scan statistics analysis of high and low IRs from 2009 to 2014.

Year	Cluster Rank	IR	Number of Districts	Districts	Number of Cases	Expected Cases	*LLR*	*RR*	*p*
2009–2011	First	High	16	All districts	13,950	12,342.71	199.20	1.28	0.001

**Table 3:** Space-time scan statistics analysis of high and low IRs from 2009 to 2014.

Year	Cluster Rank	IR	Number of Districts	Districts	Number of Cases	Expected Cases	*LLR*	*RR*	*p*
2009–2011	First	High	1	Xicheng	1892	802.80	556.71	2.46	<0.001
2012–2014	Second	Low	2	Dongcheng, Chaoyang	1714	3062.29	392.54	0.53	<0.001
2012–2014	Third	Low	2	Shijingshan, Fengtai	960	1872.19	288.03	0.49	<0.001
2009–2010	Fourth	High	4	Huairou, Miyun, Shunyi, Changping	2093	1287.08	225.05	1.68	<0.001

**Table 4:** Trajectory similarities between IR and NSPRP/NSPTBP/RSPRP/RSPTBP from 2010 to 2014.

Dist	*a* = 0.55	*a* = 0.65	*a* = 0.75	*a* = 0.85	*a* = 0.95	Average
dist_IR&NSPRP	3.07	2.87	2.68	2.50	2.35	2.70
dist_IR&NSPTBP	3.71	3.57	3.41	3.26	3.11	3.41
dist_IR&RSPRP	4.78	4.60	4.43	4.29	4.17	4.46
dist_IR&RSPTBP	5.59	5.28	5.02	4.80	4.63	5.06
